# Haemophilus influenzae type b and Streptococcus pneumoniae as causes of pneumonia among children in Beijing, China.

**DOI:** 10.3201/eid0602.000209

**Published:** 2000

**Authors:** O S Levine, G Liu, R L Garman, S F Dowell, S Yu, Y H Yang

**Affiliations:** Respiratory Diseases Branch, National Institute of Allergy and Infectious Diseases, Bethesda, MD 20892-7630, USA. OLevine@niaid.nih.gov

## Abstract

To determine if Haemophilus influenzae type b (Hib) and Streptococcus pneumoniae could be identified more often from the nasopharynx of patients with pneumonia than from control patients, we obtained nasopharyngeal swab specimens from 96 patients with chest x-ray-confirmed pneumonia and 214 age-matched control patients with diarrhea or dermatitis from the outpatient department at Beijing Children's Hospital. Pneumonia patients were more likely to be colonized with Hib and S. pneumoniae than control patients, even after the data were adjusted for possible confounding factors such as day-care attendance, the presence of other children in the household, and recent antibiotic use. In China, where blood cultures from pneumonia patients are rarely positive, the results of these nasopharyngeal cultures provide supporting evidence for the role of Hib and S. pneumoniae as causes of childhood pneumonia.


### *Haemophilus influenzae* Type b and *Streptococcus pneumoniae* as Causes of Pneumonia among Children in Beijing, China

**Orin S. Levine,*† Gang Liu,† Robert L. Garman,*‡ Scott F. Dowell,* Sangie Yu,† and Yong-Hong Yang†**
*Centers for Disease Control and Prevention, Atlanta, Georgia, USA; †Beijing Children's Hospital Affiliated with Capital University of Medical Sciences, Beijing, China; and ‡Rollins School of Public Health, Emory University, Atlanta, Georgia, USA

To determine if *Haemophilus influenzae* type b (Hib) and *Streptococcus pneumoniae* could be identified more often from the nasopharynx of patients with pneumonia than from control patients, we obtained nasopharyngeal swab specimens from 96 patients with chest x-ray-confirmed pneumonia and 214 age-matched control patients with diarrhea or dermatitis from the outpatient department at Beijing Children's Hospital. Pneumonia patients were more likely to be colonized with Hib and *S. pneumoniae* than control patients, even after the data were adjusted for possible confounding factors such as day-care attendance, the presence of other children in the household, and recent antibiotic use. In China, where blood cultures from pneumonia patients are rarely positive, the results of these nasopharyngeal cultures provide supporting evidence for the role of Hib and *S. pneumoniae* as causes of childhood pneumonia.

New vaccines for *Haemophilus influenzae* type b (Hib) and *Streptococcus pneumoniae* effectively prevent disease caused by these pathogens (1,2). However, the relatively high cost of these vaccines inhibits their widespread use in developing countries. Therefore, for most developing countries, surveillance for disease caused by Hib and *S. pneumoniae* is needed to demonstrate that the investment in these highly effective vaccines is warranted (3).

Data on the incidence of Hib and pneumococcal disease in China are limited to a few meningitis surveillance studies, which suggest a surprisingly low incidence of confirmed Hib meningitis. In most cases the rate is 5 to 25 times lower than those observed in areas of North America, Europe, Africa, South America, and Oceania where careful surveillance studies have been carried out (4-7). However, the apparent low incidence in these studies is difficult to interpret because of concerns that physicians may not routinely perform lumbar punctures on all patients with suspected meningitis, that the laboratory methods may have been inadequate, and that widespread use of oral antibiotics for outpatients may have artificially reduced the yield of cultures (8). In part because of the uncertainty surrounding the local rate of Hib disease, China has not yet made Hib vaccination a routine infant vaccination. Many authors and organizations have called for further studies of the epidemiology in China, but few data are currently available (9-11).

Pneumonia is a leading cause of illness and death among children worldwide (12). Demonstrating the role of bacterial agents such as *H. influenzae* and *S. pneumoniae* in pneumonia, however, is difficult. The most widely accepted

Address for correspondence: Orin S. Levine, Respiratory Diseases Branch, Division of Microbiology and Infectious Diseases, National Institute of Allergy and Infectious Diseases, 6700-B Rockledge Drive, Room 3133, Bethesda, MD 20892-7630, USA; fax: 301-496-8030; e-mail: OLevine@niaid.nih.gov.

method for demonstrating that a bacterial agent causes pneumonia is isolation of the bacterium from cultures of blood or, in some cases, lung aspirates. However, blood cultures are rarely positive in pediatric pneumonia even when microbiologic techniques are optimal, and fastidious organisms such as Hib will not grow unless the appropriate culture medium is used. In China, parents and physicians often resist the collection of blood for cultures, and given the high rate of prior antibiotic use, the yield from these specimens is likely to be low.

Because alternatives are needed to traditional methods of documenting Hib and *S. pneumoniae* as causes of pneumonia in China, we designed a study to investigate the use of a readily available source of specimens that is likely to be positive more often than a blood culture. We compared the rates of isolation of Hib and *S. pneumoniae* from nasopharyngeal swabs and blood cultures of patients with radiographically confirmed pneumonia and a group of control patients without pneumonia.

### The Study

The People's Republic of China is the most populous nation in the world, with an estimated population of >1.2 billion and >20 million births each year (13). Beijing, the capital, has a population of approximately 11 million people. Beijing Children's Hospital is one of the largest children's hospitals in Asia, with >700 beds in 24 wards. The outpatient department typically has 2,500 to 4,000 patient visits per day.

Before patients were enrolled, a study nurse or physician explained the study to the parent or guardian, answered questions, and obtained written informed consent. The protocol for this study was approved by the human subjects review committees at Beijing Children's Hospital and the Centers for Disease Control and Prevention.

Pneumonia cases were defined as illness in patients 2 to 60 months of age with radiographic evidence of pulmonary infiltrates and at least three of the following: fever (temperature 38.0°C), tachypnea (>50 breaths per min for infants <12 months old, and >40 breaths per min for children 12 to 60 months old), cough, auscultation findings indicative of lower respiratory disease (including rhonchi, crackles, or bronchial breath sounds), or chest indrawing.

Specimens for blood culture were collected from all 96 pneumonia patients. Urine specimens were obtained from 89 (93%) pneumonia patients and 199 (93%) controls.

Chest radiographs from all pneumonia patients were interpreted initially by a radiologist at Beijing Children's Hospital, who characterized the pattern of infiltrates as evidence of alveolar consolidation only, interstitial infiltrates only, or a mixed pattern with evidence of interstitial infiltrates and consolidation. The radiographs were then recorded by a digital camera and read later by one of the investigators who is a pediatrician (SFD). The pediatrician characterized the radiographs as having evidence of obvious or not obvious pneumonia. Both the radiologists and the pediatricians were blinded to the colonization status of the patients.

Control patients were children with a diagnosis of diarrhea or dermatitis who had no indication of respiratory tract disease. All subjects were recruited from patients attending the Outpatient Department. None of the participating children had received Hib vaccine. Of 121 eligible pneumonia patients identified in the Radiology department, 96 (79%) were enrolled; 214 controls were also enrolled, 169 with diarrhea and 45 with dermatitis. Cases and controls were frequency-matched by age in a 1-to-2 ratio in the following age categories: 2 to 5 months, 6 to 11 months, 12 to 35 months, and 36 to 60 months.

### Laboratory Methods

After measuring the distance from the nares to the earlobe, a researcher inserted a fine flexible Dacron swab past the point of mild resistance to the posterior pharynx. The swab was rotated twice, withdrawn, and immediately used to make a uniform suspension in a skim milk/glucose/glycerol solution. The suspension was then plated onto a blood agar plate with gentamicin (for isolation of pneumococci) and a chocolate agar plate supplemented with X and V factors and bacitracin (for isolation of *H. influenzae*). Plates were refrigerated before inoculation and warmed to room temperature before use.

Within 3 hours of inoculation, blood agar plates were placed in an incubator at 35°C to 37°C and 5% to 10% $CO_2$, and individual colonies were isolated in the Microbiology and Immunology Laboratory, Beijing Children's Hospital. After 18 to 36 hours of incubation, the plates were assessed for the appearance of α-hemolytic colonies resembling streptococci. A second plate was then streaked for confluent growth and an optochin disk placed on it. Strains inhibited by optochin were confirmed as pneumococci by bile solubility testing. For isolation of *H. influenzae*, X and V factor-supplemented chocolate agar plates were placed in an incubator at 35°C to 37°C and 5% to 10% $CO_2$, and single colonies were isolated. After overnight incubation, the plates were assessed each morning for 2 days for the appearance of large, flat, colorless-to-gray opaque colonies, without hemolysis or discoloration of the medium. *H. influenzae* were determined by dependency for X and V factors and absence of hemolysis on blood agar plates. All isolates were serotyped by using type-specific antiserum.

For blood cultures, which were done in the Clinical Microbiology Laboratory at Beijing Children's Hospital, 2 to 5 ml of blood was inoculated into blood culture bottles that contained agents to inhibit antibiotic activity. The bottles were incubated by the BacTec 9120 system (Organon Teknika, Durham, NC). All positive blood cultures were subcultured onto blood agar and chocolate agar plates. These plates were prepared by the same techniques and quality control used in the research laboratory at Beijing Children's Hospital. For colonies with appropriate morphologic features on subcultures, the same protocol for identification of pneumococcus and *H. influenzae* was used as described earlier. Reagents and media were checked weekly for their ability to support the growth of control strains of *H. influenzae* and *S. pneumoniae*.

Urine specimens were tested for antigens to *Legionella pneumophila* serogroup 1 by an optical immunoassay and for evidence of prior antimicrobial activity by a *Micrococcus luteus* inhibition assay. To determine if *Chlamydia pneumoniae* were present in the nasopharynx, nasopharyngeal swabs were cultured on HEp-2 cells by using a modification of standard techniques involving repeat centrifugation steps (14). *C. pneumoniae* cultures were performed only on a subset of 30 patients >2 years old.

### Statistical Analysis

Recent day-care attendance was defined as attendance in the last month before enrollment. Exposure to other children was defined as residing in the same household with at least one other child <18 years old. All analyses were conducted by SAS (SAS for Windows v.6.12; SAS Institute, Cary, NC). Logistic regression was used to assess the independent association between colonization and pneumonia and to adjust for potential confounders. Odds ratios (OR) were calculated by comparing pneumonia patients with control patients without pneumonia. Separate ORs were calculated for both the clinical and radiologic definitions of pneumonia. ORs with 95% confidence intervals that do not include 1.00 and p values <0.05 were considered statistically significant.

### Results

Four patients who were initially enrolled as pneumonia patients were later determined to have myocarditis and were thus excluded. One pneumonia patient had a blood culture positive for *S. pneumoniae*; no patients had a blood culture positive for *H. influenzae*. All urine specimens were negative for *L. pneumophila* serogroup 1. *C. pneumoniae* was not cultured from any of the 30 specimens tested. Eleven pneumonia patients were hospitalized. Among the pneumonia patients, 45 had a pattern of alveolar consolidation, 23 had an interstitial pattern, and 28 had a mixed pattern (both consolidation and interstitial).

Comparison of the patients with radiographic pneumonia and control patients showed that the distribution of ages was not consistently one pneumonia to two nonpneumonia patients in all age groups (Table 1). Although the differences in the distributions are not statistically significant, we adjusted all subsequent analyses by age to account for any residual confounding by this factor. Pneumonia patients were significantly more likely to have received antibiotics before attending the outpatient clinic, to attend day care, and to have other children in the household (Table 1; $p \leq 0.01$, for each comparison).

Of the 96 patients who had radiographic evidence of pneumonia, 32 (33%) were considered to have obvious pneumonia by the pediatrician's reading. The prevalence of Hib colonization was significantly greater among pneumonia patients than among patients without pneumonia (Table 2). The association was stronger, however, when the patients meeting the clinical definition of pneumonia were compared with the controls (OR = 5.8) than when the radiologist's definition of pneumonia was used (OR = 4.2). Multivariate analysis by logistic regression showed that the association remained after the data were

Table 1. Characteristics of pneumonia cases and diarrhea and dermatitis control patients, Beijing, China

| Risk factor | Cases (n=96) No. | % | Controls (n=214) No. | % | p-value |
|---|---|---|---|---|---|
| Age group | | | | | 0.265 |
| 2 to 5 months | 20 | 20.8 | 40 | 18.7 | |
| 6 to 11 months | 9 | 9.4 | 39 | 18.2 | |
| 12 to 35 months | 41 | 42.7 | 83 | 38.9 | |
| 36 to 60 months | 26 | 27.1 | 52 | 24.3 | |
| Daycare attendance in the past 30 days | 32 | 33.3 | 36 | 17.1 | 0.002 |
| Recent antibiotic use | 53 | 60.2 | 63 | 31.7 | 0.001 |
| At least one other child in household | 53 | 55.2 | 85 | 39.7 | 0.011 |
| Three or more persons in household | 75 | 78.1 | 154 | 72.0 | 0.254 |
| At least one current cigarette smoker in household | 67 | 69.8 | 139 | 65.0 | 0.404 |
| Currently breastfeeding | 31 | 32.3 | 89 | 41.6 | 0.120 |
| Male sex | 50 | 52.1 | 130 | 60.8 | 0.153 |
| Caregiver Education Level | | | | | 0.126 |
| No schooling | 11 | 11.5 | 10 | 4.7 | |
| Primary (1-6 years) | 17 | 17.7 | 53 | 24.8 | |
| Middle (7-9 years) | 33 | 34.4 | 82 | 38.3 | |
| Senior (10-12 years) | 22 | 22.9 | 38 | 17.7 | |
| College or higher | 13 | 13.5 | 31 | 14.5 | |

Table 2. Association of nasopharyngeal colonization with *Haemophilus influenzae* type b (Hib) and *Streptococcus pneumoniae* and radiographically confirmed pneumonia, Beijing, China

| | Controls (N=214) | Pneumonia defined by: Radiologist's reading (N=96) | | | | | Pediatrician's reading (N=32) | | | | |
|---|---|---|---|---|---|---|---|---|---|---|---|
| | No. pos. (%) | No. pos. (%) | O.R. | 95% C.I. | Adj.[a] O.R. | 95% C.I. | No. pos. (%) | O.R. | 95% C.I. | Adj.[a] O.R. | 95% C.I. |
| Hib | 4 (1.9) | 7 (7.3) | 4.23 | 1.20, 14.86 | 4.12 | 1.01, 16.80 | 3 (9.4) | 5.77 | 1.21, 27.59 | 9.62 | 1.73, 53.40 |
| *S. pneumoniae* | 83 (38.8) | 42 (43.8) | 1.22 | 0.75, 1.99 | 1.84 | 1.03, 3.29 | 16 (50.0) | 1.52 | 0.72, 3.23 | 3.03 | 1.20, 7,64 |

[a]Odds ratio adjusted for the following potential confounding factors: antecedent antibiotic use, recent day-care attendance, and other children in the household.

adjusted for potential confounders (recent antibiotic use, recent day-care attendance, and other children in the household), and in the case of the clinical definition of pneumonia, increased the strength of the association.

In univariate analysis, colonization with *S. pneumoniae* was not significantly higher among pneumonia patients than among patients without pneumonia (Table 2). This association, however, was confounded by the higher proportion of pneumonia patients with prior antibiotic use, an exposure that decreases colonization substantially. After the data were adjusted for prior antibiotic use, recent day-care attendance, and exposure to other children in the household, colonization with *S. pneumoniae* was significantly associated with pneumonia by either the radiographic or clinical definition of pneumonia.

### Conclusions

We found that Hib and *S. pneumoniae* can be isolated significantly more often from the nasopharynx of children with radiographically confirmed pneumonia in China than from comparable children without pneumonia, demonstrating the potential role of these bacteria as a cause of pneumonia in Chinese children. These results suggest that further studies should be undertaken to quantify the role of these agents in China, the prevalent serotypes, and the potential impact of vaccines for their prevention.

The prevalence rates of Hib and *S. pneumoniae* colonization observed among controls are similar to those we reported in a study of outpatients at Beijing Children's Hospital, as well as by other investigators (15,16). The prevalence of Hib colonization among nonpneumonia patients (~2%) is consistent with that observed in other studies of young children in China, but lower than commonly seen in other developing countries (5%-15%) (17-19). Even in industrialized countries such as the United States, Finland, and the United Kingdom, pre-vaccination colonization rates of 3% to 6% were generally observed (20). Host and environmental factors may play a role in this apparently low prevalence of Hib colonization in nonpneumonia patients. For example, widespread overuse of antibiotics in children and the relatively small family size as the result of the "one family, one child" policy may contribute to this observation.

The rate of positive blood cultures among pneumonia patients was lower (1%) than that generally observed in studies from other countries, but consistent with reports of a low rate of bacterial meningitis and invasive disease from mainland China and Taiwan (5,6,10). In rural Papua New Guinea, *S. pneumoniae* and *H. influenzae* were isolated from the blood of 11% and 12%, respectively, of children >6 years old with moderate or severe acute lower respiratory tract illness (21). However, a recent study from Dallas found no positive blood cultures among 106 ambulatory outpatients <5 years old with radiographically confirmed pneumonia (22). Thus, the rate of positive blood cultures was lower than that seen in hospital-based studies in developing countries but consistent with recent data from studies in industrialized countries of children with pneumonia who attend outpatient clinics.

Several steps were taken to ensure the quality of the blood specimens collected and their processing. For example, all blood specimens were collected before intravenous antibiotics were administered. The protocol for the study was to inoculate bottles with 2 and 5 ml of blood, a volume considered optimal for the 15-ml blood culture bottles used in the BacTec system, and the blood culture bottles contained resin-coated beads designed to neutralize the antibacterial activity of antecedent antibiotic use.

The observation that colonization with Hib and *S. pneumoniae* is more likely among pneumonia patients, especially those with radiographically obvious pneumonia, strengthens the contention that pneumonia is associated with these agents. This finding is consistent with the observation from studies with Hib conjugate vaccine showing that the protection afforded by these vaccines is most apparent in children with obvious radiographic pneumonia or alveolar consolidation and not apparent in children with milder forms of pneumonia (23,24). However, an inherent limitation of case-control studies is that they cannot establish that the exposure, in this case colonization with Hib or *S. pneumoniae*, preceded the onset of illness.

The potential role of other agents of pneumonia besides Hib and *S. pneumoniae* remains unclear. In this study, *H. influenzae* colonization (regardless of serotype) was also associated with pneumonia (data not shown). The observation that no patients had antigens to *L. pneumophila* serogroup 1 in urine is consistent with findings from the United States and elsewhere indicating that this agent rarely causes pneumonia among young children (25). The finding that none of 30 patients >2 years of age were positive for *C. pneumoniae* by culture suggests that if this organism causes pneumonia in this age group, alternative methods for detection are needed to define its role. The potential role of other agents such as *Mycoplasma pneumoniae* and viruses such as respiratory syncytial virus warrants further investigation. Characterizing the epidemiology of these agents may have important implications for the choice of empiric therapy in pneumonia patients and for the development of vaccines for pneumonia prevention.

### Acknowledgments

We thank the following persons for assistance with laboratory training and specimen processing: Barry Fields, Jan Pruckler, Steve Bosshardt, Valerie Stevens, John Elliott, and Sophie Newland at CDC, and Jie Li, Yue Juan Tong, Wei Gao, Yan Li, and Gui Rong Zhang at Beijing Children's Hospital. We also thank Sun Guo Qiang, Jin Jin Zeng, and Sai Ying Xu, the radiologists who systematically interpreted the radiographs for us in Beijing, and Zaifang Jiang and Dexiu Yao for their support and assistance.

This work was supported by funding from the Emerging Infectious Diseases Program, National Center for Infectious Diseases, Centers for Disease Control and Prevention.

Dr. Levine is an epidemiologist in the Division of Microbiology and Infectious Diseases, National Institute of Allergy and Infectious Diseases. His research interests include bacterial pneumonia and meningitis and their prevention by vaccination. He collaborates on related projects in South America, Africa, and the Middle East.

### References

1. Steinhoff MC. *Haemophilus influenzae* type b infections are preventable. Lancet 1997;349:1186-7.
2. Eskola J, Anttila M. Pneumococcal conjugate vaccines. Pediatr Infect Dis J 1999;18:543-51.
3. Levine MM, Levine OS. Disease burden, public perception and other factors that influence new vaccine development, implementation and continued use. Lancet 1997;350:1386-92.
4. Yang Y, Leng Z, Shen X, Lu D, Jiang Z, Rao J, et al. Acute bacterial meningitis in children in Hefei, China 1990-1992. Chinese Medical Journal 1996;109:385-8.
5. Wang CH, Lin TY. Invasive *Haemophilus influenzae* diseases and purulent meningitis in Taiwan. J Formos Med Assoc 1996;95:599-604.
6. Lau YL, Low LC, Yung R, Ng KW, Leung CW, Lee WH, et al. Invasive *Haemophilus influenzae* type b infections in children hospitalized in Hong Kong, 1986-1990. Hong Kong Hib Study Group. Acta Paediatrica 1995;84:173-6.
7. Levine OS, Schwartz B, Pierce NF, Kane M. Development, evaluation and implementation of Hib vaccines for young children in developing countries: current status and priority actions. Pediatr Infect Dis J 1998;17:S95-113.
8. Salisbury DM. Summary statement: The First International Conference on *Haemophilus influenzae* type b infection in Asia. Pediatr Infect Dis J 1998;17:S93-5.
9. Lau YL, Yung R, Low L, Sung R, Leung CW, Lee WH. *Haemophilus influenzae* type b infections in Hong Kong. Pediatr Infect Dis J 1998;17:S165-9.
10. Yang Y, Shen X, Jiang Z, Liu X, Leng Z, Lu D, et al. Study on *Haemophilus influenzae* type b diseases in China: the past, present and future. Pediatr Infect Dis J 1998;17:S159-65.
11. Lee HJ. Epidemiology of systemic *Haemophilus influenzae* disease in Korean children. Pediatr Infect Dis J 1998;17:S185-9.
12. World Health Organization. Acute respiratory infections: a forgotten pandemic. Bull WHO 1998;76:101-3.
13. UNICEF. The state of the world's children 1998. New York: Oxford University Press; 1999. p. 1-131.
14. Pruckler J, Masse N, Stevens V, Gang L, Yang Y, Zell ER, et al. Optimizing culture of *Chlamydia pneumoniae* using multiple centrifugations. J Clin Microbiol. In press 1999.
15. Whitney CG, Wang JF, Elliott JA, Levine OS, Dowell SF, Yang YH. Nasopharyngeal carriage of *Streptococcus pneumoniae* among children in Beijing: drug resistance and risk factors for carriage. 63-63. 1998. Washington: World Health Organization. Pneumococcal vaccines for the world 1998 conference. 10-12-1998.
16. Wang H, Huebner R, Chen M, Klugman K. Antibiotic susceptibility patterns of *Streptococcus pneumoniae* in China and comparison of MICs by agar dilution and E-test methods. Antimicrob Agents Chemother 1998;42:2633-6.
17. Sung RY, Ling JM, Fung SM, Oppenheimer SJ, Crook DW, Lau JT, et al. Carriage of *Haemophilus influenzae* and *Streptococcus pneumoniae* in healthy Chinese and Vietnamese children in Hong Kong. Acta Paediatrica 1995;84:1262-7.
18. Bijlmer HA, Evans NL, Campbell H, van Alphen L, Greenwood BM, Valkenburg HA, et al. Carriage of *Haemophilus influenzae* in healthy Gambian children. Trans R Soc Trop Med Hyg 1989;83:831-5.
19. Gomez E, Moore A, Sanchez J, Kool J, Castellanos PL, Feris JM, et al. The epidemiology of *Haemophilus influenzae* type b carriage among infants and young children in Santo Domingo, Dominican Republic. Pediatr Infect Dis J 1998;17:782-6.
20. Barbour ML. Conjugate vaccines and the carriage of *Haemophilus influenzae* type b. Emerg Infect Dis 1996;2:176-82.
21. Barker J, Gratten M, Riley I, Lehmann D, Montgomery J, Kajoi M, et al. Pneumonia in children in the Eastern Highlands of Papua New Guinea: a bacteriologic study of patients selected by standard clinical criteria. J Infect Dis 1989;159:348-52.
22. Wubbel L, Muniz L, Ahmed A, Trujillo M, Carubelli C, McCoig C, et al. Etiology and treatment of community-acquired pneumonia in ambulatory children. Pediatr Infect Dis J 1999;18:98-104.
23. Mulholland K, Hilton S, Adegbola R, Usen S, Oparaugo A, Omosigho C, et al. Randomized trial of *Haemophilus influenzae* type b tetanus protein conjugate for prevention of pneumonia and meningitis in Gambian infants. Lancet 1997;349:1191-7.
24. Levine OS, Lagos R, Munoz A, Villaroel J, Alvarez AM, Abrego P, et al. Defining the burden of pneumonia in children preventable by vaccination against *Haemophilus influenzae* type b. Pediatr Infect Dis J 1999;18:1060-4.
25. Orenstein WA, Overturf G, Leedom JM, Alvarado R, Geffner M, Fryer A, et al. The frequency of *Legionella* infection prospectively determined in children hospitalized with pneumonia. J Pediatr 1981;99:403-6.
